# Clinical Implication of Phosphodiesterase-4-Inhibition

**DOI:** 10.3390/ijms23031209

**Published:** 2022-01-21

**Authors:** Martin Alexander Schick, Nicolas Schlegel

**Affiliations:** 1 Department of Anesthesiology and Critical Care, Medical Center—University of Freiburg, 79106 Freiburg, Germany; 2 Faculty of Medicine, University of Freiburg, 79110 Freiburg, Germany; 3 Department of General, Visceral, Transplant, Vascular and Pediatric Surgery, University Hospital Wuerzburg, 97080 Würzburg, Germany; schlegel_n@ukw.de

**Keywords:** phosphodiesterase, phosphodiesterase-4, phosphodiesterase-inhibitors, PDE, PDE4-I

## Abstract

The pleiotropic function of 3′,5′-cyclic adenosine monophosphate (cAMP)-dependent pathways in health and disease led to the development of pharmacological phosphodiesterase inhibitors (PDE-I) to attenuate cAMP degradation. While there are many isotypes of PDE, a predominant role of PDE4 is to regulate fundamental functions, including endothelial and epithelial barrier stability, modulation of inflammatory responses and cognitive and/or mood functions. This makes the use of PDE4-I an interesting tool for various therapeutic approaches. However, due to the presence of PDE4 in many tissues, there is a significant danger for serious side effects. Based on this, the aim of this review is to provide a comprehensive overview of the approaches and effects of PDE4-I for different therapeutic applications. In summary, despite many obstacles to use of PDE4-I for different therapeutic approaches, the current data warrant future research to utilize the therapeutic potential of phosphodiesterase 4 inhibition.

## 1. Introduction

A milestone in the discoveries of cellular signaling was the identification of the second messenger, 3′,5′-cyclic adenosine monophosphate (cAMP), by Earl Sutherland et al., 1957. Cyclic AMP signaling is compartmentalized within the cell, which explains how this single second messenger can have a plethora of different and sometimes even opposing effects, making it a key player of different cellular functions within the body [1]. cAMP is generated by adenylyl cyclase (AC) after activation of G protein-coupled receptors (GPCR) [2]. The spatial and temporal control of cAMP degradation within cells is regulated by phosphodiesterases (PDEs) [3].

The identification of phosphodiesterase (PDE) enzymes nearly 60 years ago was important, since the pharmacologic inhibition of PDE offers an enormous potential of therapeutic options for many different diseases. Currently, 11 different PDEs are known, which have different subtypes. They are the only superfamily of enzymes with the ability to convert cyclic purine nucleotides—3′,5′-cyclic adenosine monophosphate (cAMP) and 3′,5′-cyclic guanosine monophosphate (cGMP)—to AMP and GMP, respectively. It is known that PDE 1-3 and 10-11 degrade both cAMP and cGMP, whereas PDE 5, 6 and 9 only degrade cGMP, and PDE 4, 7 and 8 degrade only cAMP [4].

Many experimental and clinical approaches have uncovered that phosphodiesterase-4-inhibition bears enormous therapeutic potential for different pathological conditions. Based on PDE4 tissue expression patterns within the central nervous and immune systems, over thirty years ago researchers speculated that the phosphodiesterase-4-inhibitor rolipram may have the potential to treat depression and asthma [5,6]. In the following years, many other diseases, including atopic dermatitis, psoriasis, COPD, rheumatoid arthritis, multiple sclerosis, inflammatory bowel disease, premature birth (tocolysis), schizophrenia, allergies, autoimmune diseases, dementia and stroke were added to the list of potential therapeutic PDE4-I targets [7,8]. The aim of this narrative review is to summarize the most important findings and recent developments of phosphodiesterase-4-inhibition as a promising target for future pharmacological therapeutic intervention. In the first part of the review, we will briefly introduce the most important basic principles of cAMP-dependent signaling and phosphodiesterase-4-inhibition; in the second part we will focus on the therapeutic potential in different organs.

### 1.1. Basic Principles of cAMP-Dependent Signaling and Phosphodiesterase-4-Inhibition

**Figure 1:**
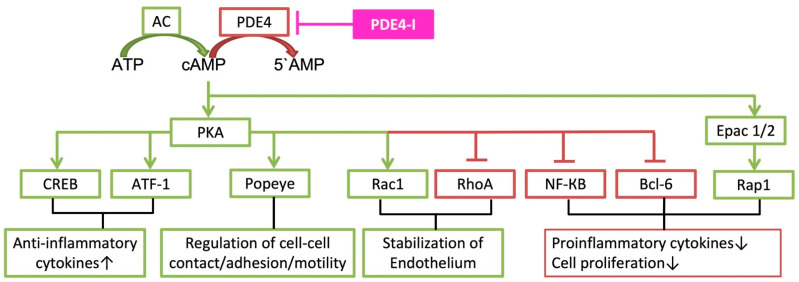
Description of cAMP cascades, which are involved in clinical implications of phosphodiesterase-4-inhibition. ATP (adenosine triphosphate); AC (adenylyl cyclase); cAMP (3′,5′-cyclic adenosine monophosphate); PDE4 (phosphodiesterase-4); PDE4-I (phosphodiesterase-4-inhibitor); 5’AMP (5′-adenosine monophosphate); PKA (protein kinase A); Epac 1/2 (exchange protein directly activated by cAMP 1 and 2); CREB (cAMP response element binding protein); ATF-1 (cAMP-dependent activating transcription factor 1); Popeye (Popeye domain family Rac1 (Ras-related C3 botulinum toxin substrate 1)); RhoA (Ras homolog family member A); NF-κB (nuclear factor ‘kappa-light-chain-enhancer’ of activated B-cells); Bcl-6 (B-cell lymphoma 6 protein); Rap 1 (Ras-related protein 1).

The two main signaling axes downstream of cAMP are the activation of protein kinase A (PKA) and exchange proteins directly activated by cAMP (Epac). These downstream cascades are included in pro- and anti-inflammatory cytokine release (see Figure 1) [9]. In addition, cAMP acts downstream by cyclic nucleotide-gated ion channels and the Popeye domain family [10,11,12,13]. The latter are involved in the regulation of epithelial cell-cell contact, where they regulate cell-cell adhesion and motility, especially in cancer (for details see [12]). Cyclic nucleotide-gated ion channels can be activated by cyclic AMP, which results in their opening and to cAMP-induced calcium influx in cells. Therefore, cAMP has pleiotropic effects via these channels in different cells. PKA, Epac 1/2 and cyclic nucleotide-gated ion channels are activated by elevated levels of cAMP [14,15]. When cAMP binds to PKA, PKA releases the catalytic PKA subunits to phosphorylate many targets. One of these targets is the cyclic AMP response element-binding protein (CREB), which acts as a transcription factor to cAMP response element (CRE) [16]. If CREB is activated and CREB-binding protein is recruited, the transcription of the target gene is initiated [16]. In addition, ATF-1 (cAMP-dependent activating transcription factor 1) is regulated by cAMP and PKA [17]. These two axes of transcription are involved in the upregulation of anti-inflammatory cytokines. The stabilization of the endothelium is driven by PKA-dependent signaling leading to activation of the small GTPase Rac1 (Ras-related C3 botulinum toxin substrate 1), which is paralleled by RhoA (Ras homolog family member A) inactivation (see Section 2.5). Another target of PKA is NF-κB (nuclear factor ‘kappa-light-chain-enhancer’ of activated B-cells), which induces inflammatory gene expression, such as IL-1ß, IL-6 or TNF-α. The cyclic AMP/PKA cascade inhibits NF-κB in most cell types and therefore reduces proinflammatory cytokine [4].Cell proliferation as well as proinflammatory cytokines can be induced by Bcl-6 (B-cell lymphoma 6 protein). PKA can inhibit Bcl-6 via ERK 1/2 [18]. Decrease of inflammatory gene transcription can also be regulated with cAMP by Epac 1/2, which activates the transcription protein Rap 1 (Ras-related protein 1).

**Figure 2:**
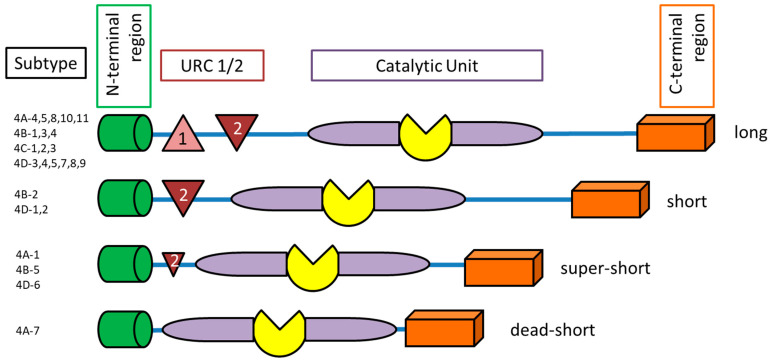
Schematic structure of phosphodiesterase-4 isoforms. UCR (upstream conserved region).

PDE4 has four subtypes (A, B, C and D). These subtypes are encoded on separate genes. Each subgroup has an additional 3–11 different isoforms, which have a unique N-terminal end (Figure 2). The N-terminal end can further be classified into the following groups: dead-short, super-short, short, and long-form. The upstream conserved region (UCR) exists in two variants (UCR1 and UCR2). The UCR clusters UCR1 and 2 are only located on the long-form of PDE4 variants [3,19], and the short-form contains UCR2 and the super-short truncated UCR2 [3]. UCR1/2 have a key regulatory function of the catalytic unit of PDE4. UCR1 has a phosphorylation site of cAMP-dependent protein kinase A (PKA) and UCR2 may have an inhibitory effect on the PDE4 catalytic unit and interact with UCR1 [20]. Therefore, the different UCR1/2 regions and distribution have a regulatory pattern for PDE4 subfamilies. Only PDE4B, C and D have the extracellular signal-regulated kinase (ERK)-phosphorylation subunits, which are inserted in the catalytic PDE4 unit. The phosphorylation of the ERK-units inhibits hydrolytic activity of the long PDE4 form, has weak or no effect on the super-short form and can increase hydrolytic activity of the short PDE4 form.

Furthermore, there are two conformational states of PDE4: high-affinity and low-affinity rolipram-binding states, the HARBS and LARBS [21]. HARBS are exclusively located in the brain tissue and LARBS are present both in the brain and peripheral tissues.

### 1.2. Pharmacological Agents for Therapeutic Phosphodiesterase Inhibition

In general, PDE4-inhibitors (PDE4-I) can be divided into three structural classes: (I) structural analogues of rolipram, (II) structural analogues of nitraquazone and (III) structures related to the xanthine nucleus [22].

## 2. Therapeutic Approaches of Phosphodiesterase-4-Inhibition in Different Organs

**Figure 3:**
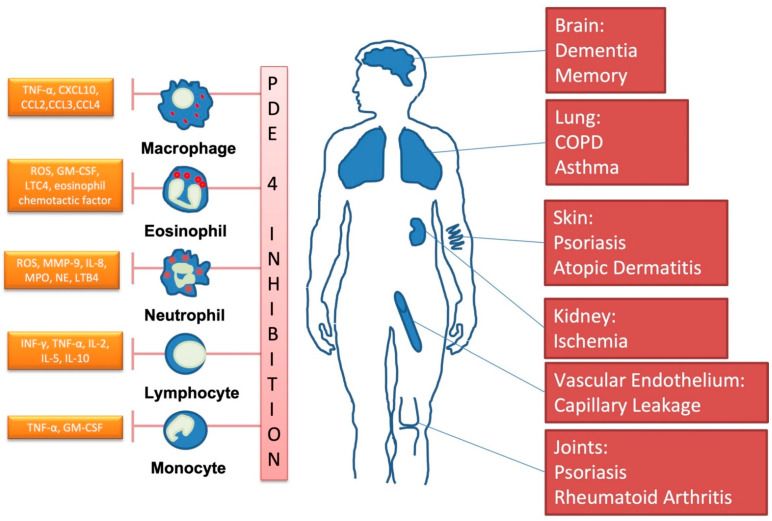
Important clinical targets of phosphodiesterase-4-inhibition. (Own drawing, modified based on [23]).

Many organs, including the immune system, and diseases can be targeted by PDE4-inhibitors, as summarized in Figure 3.

### 2.1. The Central Nervous System

Similar patterns of distribution of PDE4 are found across the brains of rats, primates and humans (for detailed review see Zhang [24]). PDE4 is highly expressed in the frontal cortex, hippocampus, olfactory bulb (which are important for modulation of antidepressant actions) and cerebellum [24]. Of note, AC (synthesis of cAMP) is primarily located in dopamine-rich brain areas, such as the striatum, nucleus accumbens and substantia nigra [25,26]. PDE4A and D are mainly expressed in the cerebral cortex, olfactory bulb, hippocampal formation and brain stem [24]. PDE4B is found in the striatum, amygdala, hypothalamus, thalamus, frontal cortex, olfactory bulb and is the only PDE4 which is located in the white matter of brain [3,24]. PDE4A, B and C can be found in neurons, and PDE4C is only located in peripheral tissue. Therefore, it is not surprising that PDE4A and D play an important role in the mediation of antidepressant effects and memory function, whereas PDE4B is involved in stress- and dopamine-associated processes, such as anxiety, schizophrenia and psychosis [24,27]. However, the noradrenergic axis of antidepressant effects may also involve PDE4B, because this is the only PDE4 in the noradrenergic-rich neurons of the locus coeruleus, and PDE4B knockout mice showed no response to desipramine [24].

The cyclic-AMP cascades underpin critical pathways necessary for brain development and function [27]. Thus, the “cAMP theory of depression” describes that cAMP signaling is downregulated in unmedicated major depressive disorder (MDD) patients and increased by treatment (e.g., by selective serotonin reuptake inhibitors (SSRI)). Chronic administration of antidepressants increases PDE4 activity as well as levels of PDE4 mRNA and protein, which can be interpreted as a response to upregulated cAMP [28,29,30]. Thus, postmortem studies in human individuals with untreated depressive disorders revealed an overall decrease in cAMP-cascade activity. Furthermore, disturbances of cAMP pathways are also shown in Alzheimer‘s disease, tardive dyskinesia and multi-infarct dementia [31].

The potent antidepressant effect of rolipram was first found in 1983 in animal experiments. As mentioned above, there are two conformational states of PDE4: high-affinity (HARBS) and low-affinity rolipram-binding (LARBS) [21]. The differential distribution in the brain revealed different effects with regard to antidepressant medication. HARBS are exclusively located in the brain tissue, mainly in the hippocampus, frontal cortex and olfactory bulb, whereas LARBS are present in both the brain and peripheral tissues. These cerebral “HARBS” regions are also targets for other antidepressant medication. In animal studies, repeated treatment with antidepressant drugs (desipramine and fluoxetine) increased HARBS, but not LARBS [32]. In addition, application of rolipram with high affinity to HARBS had the best antidepressant results compared to CDP840 (PD-4-I), which has a high affinity to LARBS and showed the worst results. Thus, the distribution of the different conformational states may play a pivotal role in depression and its therapy [28,29,30]. Extracellular signal-regulated kinase (ERK)-cascade is increased by antidepressant therapy. Therefore, it is important to know that ERK-phosphorylation subunits are inserted in the catalytic PDE4 unit with different effects.

The prevalence of age-related cognitive dysfunction has grown with increased life expectancy [33]. The need for successful, targeted treatment remains very high. Cyclic AMP response element binding protein (CREB) is a transcription factor activated by PKA phosphorylation [34]. PKA is activated by high cAMP levels. Animal studies revealed that CREB deficiency or an increase of CREB-binding protein reduced memory storage [3,35]. All three of cAMP, PKA and CREB have been shown to play a pivotal role in memory storage and synaptic plasticity [3]. Therefore, inhibition of PDE4 in order to improve cognitive function was evaluated in young, healthy human. Administration of 100 µg of roflumilast (selective PDE4-I for COPD treatment) improved early information processing, which is known to be impaired in Alzheimer’s disease [36]. Furthermore, the same dosage of roflumilast (100 μg) improved the delayed recall performance of the participants (60–83 y) [37]. Both studies were designed as “acute application“ of roflumilast with a fifth dosage of COPD therapy. Increased doses up to 1000 µg roflumilast showed typical adverse side effects of PDE4-I: headache, dizziness, insomnia and diarrhea. These side effects, with the addition of nausea and emesis (due to HARBS [38]) during chronic treatment with a PDE4-I (in this case, rolipram) prevent their use for long-term treatment (e.g., for depression).

Thus, PDE4 distribution in the brain, disregarding subcellular compartmentalization, is a complex orchestra of cAMP regulation. This melody is not yet fully explored, and therefore the understanding of the function, regulation and distribution of the specific PDE4 subtypes may offer the development of specific inhibitors to create a mechanism-based therapeutic approach.

### 2.2. The Lungs 

In the lungs, cAMP and cGMP are involved in cell proliferation, migration, differentiation, remodeling, secretion of inflammatory cytokines, tone of smooth muscle cells and stabilization of the endothelial and epithelial barriers [2,39,40,41]. In lung tissue, cAMP and cGMP are acting downstream by cyclic nucleotide-gated ion channels, PKA, cGMP-dependent protein kinase (PKG), exchange proteins directly activated by cAMP (Epac) and through the Popeye domain family [10,11,12,13].

Interestingly, the extensive description of the distribution of PDE isoforms with different subunits, as mentioned above in the brain, is missing for the lung tissue and cells. However, PDE4A (along with PDE4A1) has been detected in inflammatory cells, fibroblasts and pulmonary artery smooth muscle (PASM) in the lungs [42,43,44,45]. PDE4B is highly expressed in the inflammatory cells, and is therefore also seen in the lungs, with the exception of PDE4B5 (the brain-specific isoform) [46,47]. PDE4C is absent in lymphocytes, neutrophils and eosinophils, and also has not been described to play a role in asthma or COPD [48,49]. It is worth noting that PDE4D isoforms show a high different-tissue-distribution pattern between species. All isoforms are found in humans, but, e.g., PDE4D7 is highly expressed in the human lung but not seen in mice and rats [50]. Furthermore, PDE4-D6 and PDE4-D4 expression levels are relatively low in human lungs [51].

COPD and asthma affect at least 300 million people worldwide. About 461,000 patients die annually due to asthma worldwide [52]. The cost of about USD 1000 per year per asthma patient is another reason why development of effective drugs with new therapeutic targets has been, and still is, a relevant task [50,53]. As mentioned above, asthma was one of the first foci in the development of PDE-I for clinical use. The bronchodilation effect is preferentially mediated via PDE4D, whereas the anti-inflammatory effect is via PDE4B in mice [54] and might act through PDE4A (making a case for cilomilast) in humans [38,55,56].

COPD and asthma are characterized by airway obstruction. Both entities are distinguished by inflammation: COPD by neutrophils, macrophages and CD8+ T-lymphocytes; asthma by CD4+ T-lymphocytes, mast cells and eosinophils [50,53,57,58]. However, asthma-related bronchoconstriction is reversible by bronchodilators, which is not the case in COPD [59,60,61]. Theophylline, a PDE-I, is still used, but not recommended for all patients due to its narrow tolerability margin, with severe side effects such as high pharmacological interaction with the CYP1A2 enzyme [38]. However, roflumilast, a PDE4-I, has been used since 2011 as a supplemental treatment for COPD with frequent exacerbations. Roflumilast or other selective PDE4-I are not yet been recommended for patients with asthma due to insufficient evidence and controversial results from different trials [52,62,63].

Regarding COVID-19, it has been suggested that PDE4-I may be helpful to improve therapeutic efforts [64,65,66]. However, to date this remains speculative, and to our knowledge, no clinical trial to treat COVID-19 using PDE4-I is currently underway.

### 2.3. The Skin

Atopic dermatitis (AD) and psoriasis (PS) are chronic inflammatory skin diseases. AD affects up to 20% of children, and 2–3% of the world population suffers from PS (around 125 million). Both diseases result in systemic (not only in the skin) inflammation, with increase leucocytes, lymphocytes and proinflammatory cytokines. Interestingly, the Th1- and Th17-pathways are involved in PS, whereas Th2 is predominant in AD. AD and PS have a complex pathological interplay between decreased epithelial skin barrier, genetic predisposition, immune dysregulation and environmental triggers. Along with increased prevalence of sleep dysregulation, stroke and asthma, AD patients have a high rate of psychiatric diagnoses, such as depression and anxiety,. Psoriasis is associated with rheumatological and cardiovascular diseases, COPD and mental health syndromes (depression, suicidal ideation and alcohol abuse) [67]. The treatment of both is limited to topical therapy and unspecific immunosuppressants with poor tolerability and low efficacy. New treatments must be developed due address the absolute reduction in quality of life of AD and psoriasis patients. It must be emphasized that it is not clear whether these diseases are causally linked to alterations of PDE4-dependent pathways. Rather, we believe that the inflammatory response observed in these diseases may be critically linked to PDE4-signalling.

#### 2.3.1. Psoriasis

The expression of different PDE4 isoforms is increased in PS skin (also AD and discoid lupus erythematosus) compared to healthy individuals. PDE4-I can reduce the number of T-cells, NK-cells and CD11c myeloid dendritic cells in the dermis and epidermis of PS patients [68,69]. Inhibition of PDE4 showed downregulation of the plasma levels of TNF-α, IL-17F, IL-17A and 22 [70]. Expression of the PDE4A subtype increased in nearly all cell types of the skin of PS patients. PDE4B is present in vessels and in immune cells, whereas PDE4D is expressed in fibroblasts and endothelial cells [71].

Apremilast has been approved orally for treatment of moderate-to-severe PS. IL-17 is the most important predictor for PS therapy improvement [70]. Furthermore, IL-10 gene expression increased only in the skin of patients who responded to apremilast, which can be interpreted as an anti-inflammatory response. Oral intake showed sufficient therapy results, but with the commonly known side effects of diarrhea (7.7%%), nausea (8.9%) and headache (5.9%) [72]. Furthermore, extended treatment with apremilast can lead to depressed mood or depression [23]. Therefore, a topical application was tested in a phase 2b double-blind trial with 331 patients. A total of 113 patients received 0.15% roflumilast, 109 received 0.3% roflumilast and 109 patients only the vehicle. After 8 weeks, 90% of patients with at least mild intertriginous psoriasis who had been treated with 0.3% roflumilast topically showed treatment success, with less than 1% exhibiting side effects (such as nausea and diarrhea) [73]. Interestingly, there was an increase (6–7%) in upper respiratory infections in the roflumilast compared to vehicle cream.

#### 2.3.2. Atopic Dermatitis

In pediatric care, there is a need for long-term treatment of AD, as long-term immunosuppressant therapy is unfeasible due to adverse side effects. AD is most likely a TH-2 type dominated disease, with epidermal barrier dysfunction, intense pruritus, eczematous lesions and skin inflammation. The impaired skin barrier triggers inflammation by allowing access to microbes, irritants and allergens. Increased numbers of TH2, TH22 and TH17 cells are observed both in the skin lesion and in other areas. It is known that AD is characterized by a TH1/TH2 imbalance and increased level of IL-4, IL-5, IL-13, IL-25, IL-31 (itching mediator, also thymic stromal lymphopoietin [74]) and IL-33; CCL17, CCL18, CCL22 and CCL26 were also observed in AD skin lesions. For the TH17 axis, upregulation of IL-17, IL-17a, CXCl1, CCL20 and elafin (PI3) were found in chronic and acute AD (for details see [75]).

Compared to healthy individuals, lymphocytes of AD patients showed increased PDE4 activity. These effects were even observed when AD had been in remission for up to 20 years [76]. Fibroblasts of AD skin lesions all showed increased PDE4 subfamilies (A, B, C and D). AD patients who were treated with apremilast orally showed improvement of AD but had an incidence of 90% nausea (30 mg/d) [77]. Crisaborole ointment is a PDE4-I (with less inhibitory effects to PDE1, PDE3 and PDE7), which is approved for topical skin treatment of mild to moderate AD. Crisaborole has a low systemic absorption rate and is quickly metabolized to its inactive form [78]. In cell cultures (human peripheral blood mononuclear cells, human monocytes and monocyte-derived dendritic cells), crisaborole reduced the release of IL-4,-5,-13,-17 and -23, TNF-α and INF-γ [74]. In a phase 2a intrapatient double-blind study with punch biopsies at baseline, day 8 and 15, crisaborole was effective in downregulation of epidermal proliferation markers KRT16, CCL17, CCL22, PI3/elafin, S100A12, IL-13, CCL18, MMP12, IL-10, thymic stromal lymphopoietin receptor and IL-31. No changes in TH2-related genes, such as IL-5 or CCL13 or -26 were observed. Th1-related cytokines IL-5 and -15 were also downregulated by topical crisaborole, as well as IL-12/IL-23 p40, CXCL9 and -10, INF-γ and IL-9 (for details see [79]). Thus, PDE4-I improved lesion signs and symptoms, skin barrier function and epidermal hyperplasia and proliferation with modulation of the TH2 and TH17/TH22 axes [79]. For the treatment of AD, other topical PDE4 inhibitors are currently under investigation, including DRM02, E6005/RVT-501, LEO 29102 and OPA-15406/MM36.

### 2.4. The Kidney

The global prevalence of chronic kidney failure is 242 cases per million people. This number increases annually by about 8% [80]. Thus, chronic and acute kidney failure is a public health problem. In diabetic kidney disease, the change of vascular structure creates a state of hypoperfusion in the renal parenchyma, which induces interstitial fibrosis and glomerular sclerosis. Renal interstitial fibrosis leads to the activation of fibroblasts and accumulation of extracellular matrix proteins, which ends in the loss of kidney function and end stage kidney disease [81].

In deoxycorticosterone acetate-induced hyperglycemia, represented by a murine hypertension kidney failure model, mRNA expression was elevated for PDE4A, B and D in the kidney after three weeks [82]. Compound A, a selective PDE4-I (*N*-[Amino (dimethylamino)methylidene]-4-[(3aS,9bR)-8-ethoxy-7-methoxy-1,3,3a,9b-tetrahydrofuro[3,4-c]isoquinolin-5-yl]benzamide), significantly suppressed the urinary albumin creatinine ratio, urinary KIM-1 (kidney injury molecule) and MCP-1 (monocyte chemoattractant protein) levels. Furthermore, renal mRNA expression of profibrotic genes, including TGF-β, collagen 1A1 and fibronectin, were significantly decreased by compound A. This might be mediated through the strong antifibrotic and anti-ROS effects of PDE4 inhibitors. In TGF-β-stimulated human mesangial cells, compound A significantly decreased CTGF, PAI-1, collagen 1A1 and fibronectin mRNA. The antifibrotic effects of compound A in these cells may partially be derived from the suppression of Smad2 phosphorylation. Furthermore, compound A showed antifibrotic effects with podocytes and renal epithelial cells in vitro.

Cisplatin, a widely used chemotherapeutic agent, can induce nephrotoxicity. In a mouse model of cisplatin-induced AKI, cisplatin upregulated mRNA expression of PDE4B and D but did not affect the A and C subtypes [83]. Additionally, only PDE4B protein expression (as well as staining of this subtype in kidney tissue) was upregulated. Cilomilast significantly improved cisplatin-induced AKI by reducing renal pathological damage and renal tubular injury. Reduction of mRNA levels of IL-1, IL-6, TNF, MCP-1, nucleotide-binding oligomerization domain-like receptor protein 3 (NLRP3) and serum IL-6 were achieved by cilomilast treatment. The effect of cilomilast was only partly seen by knock-out of PDE4B in this model, and, therefore, PDE4B may be more important in the cascade of cisplatin induced AKI than other PDE4 subtypes. Additional application of cilomilast rescued the phosphorylation of AKT and blunted the reduction of PI3K.

Renal ischemia is a common problem following surgical procedures such as transplantation, partial nephrectomy or cardiac arrest. Very little is known about PDE4 under these conditions. One animal trial showed protective effects of renal ischemic reperfusion injury by rolipram. The most pronounced effect was seen when PDE4-I was administered 30 min after reperfusion (renal artery clamping for 30 min) [84]. However, the mechanism is unclear. In an ECLS-rat model (extra-corporal life support) with cardiac arrest, we showed that continuous IV application of PDE4-I after ROSC effectively reduced the kidney histopathology injury score and improved kidney function (measured by decreased serum creatinine and NGAL-levels) [85].

Another major killer of renal function is sepsis. In a CLP-mouse model, roflumilast alleviated sepsis-induced AKI as revealed by reductions in BUN, creatinine, NGAL, KIM-1, IL-1b, TNF-a and IL-6. Furthermore, the inflammasome was attenuated in roflumilast-treated CLP-mice [86]. These results may be explained by decreased oxidative renal damage, as seen by the reduction of MDA and NO in renal tissue when rolipram was applied daily in *E. coli*-induced pyelonephritis [87]. In a CLP-rat sepsis model, rolipram also had a u-shaped dose pattern (0.3–10 mg/kg IP rolipram) to restore renal microcirculation and blood flow and reduce cellular stress [88]. Rolipram showed effects on the cardiovascular system, as revealed by increased heart rate and decreased blood pressure at some time points. In this trial, PDE4-I stabilized endothelial barrier function.

### 2.5. The Vascular Endothelial Barrier

cAMP is one of the most potent signaling molecules for stabilization of the endothelial barrier (EB), both under resting conditions as well as when challenged by barrier-destabilizing mediators. It is important to note that increased cAMP levels in endothelial cells can have opposing effects, depending on where in the cell cAMP signaling takes place [89]. The two main signaling axes for inducing endothelial barrier stabilization downstream of cAMP include activation of protein kinase A (PKA) as well as Epac and its effector GTPase, Rap1; further downstream both axes merge to Rac1 [90]. The appropriate localization of PKA to mediate downstream signaling in endothelial cells is important [91].

Meanwhile it is well-established that inflammation induces loss of endothelial cAMP levels, which contributes significantly to loss of endothelial barrier function [90,92,93]. In line with this observation, there is evidence that that the intravenous application of the PDE4-inhibitors rolipram and roflumilast attenuates microvascular leakage, leading to improved microcirculation in different animal models of systemic inflammation and sepsis [92,94,95]. Importantly, the beneficial effects of PDE4-inhibitors appear to be predominately mediated by direct effects on endothelial cells, since little change in cytokine expression or macrohemodynamics were observed [92,94]. This makes pharmacological PDE4-inhibition an interesting and promising option in sepsis therapy. However, this has not been attempted in humans yet, due to the fact that no formulation for IV application of PDE4-inhibitors is available.

### 2.6. Inflammation

Cyclic-AMP signaling has a pivotal role in modulating the inflammatory response in patients. The signaling pathways are included in pro- and anti-inflammatory cytokine release, antigen presentation, T-cell activation and neutrophil degranulation. In the following, we will briefly describe the most important cAMP-dependent mechanisms on immune cells.

#### 2.6.1. Neutrophils

In various LPS models (mice, rats), PDE4-I lead to decreased neutrophil recruitment [39,96]. In addition, the release of proinflammatory mediators by neutrophils, MMP-9 (matrix metalloproteinase) [97], neutrophil elastase [98], myeloperoxidase, ROS (reactive oxygen species) and leukotriene B4 [99] is decreased following PDE4-I exposure. Neutrophil infiltration and accumulation were decreased by PDE4 inhibition in a cigarette smoke (CS) COPD model [100]. Interestingly CS may reduce the anti-inflammatory effect of cAMP, because Epac1, but not Epac2, expression is downregulated by CS in PASM and lung tissue from COPD patients [101]. Furthermore, roflumilast and cilomilast reduced IL-8, TNF-α, GM-CSF, neutrophils and eosinophils in the sputum of COPD patients.

#### 2.6.2. Eosinophils

A large body of evidence from various models showed that PDE4-I also decreased eosinophil infiltration into the lungs. PDE4-I [50] can diminish eosinophil degranulation induced by GM-CSF, platelet activating factor or chemotaxis. Interestingly, PDE4B knock-out mice showed reduced eosinophil recruitment and were not able to develop hyperresponsiveness in an allergen-induced airway model [102], whereas PDE4-D deficient mice developed normal eosinophil infiltration into the lung tissue [47].

#### 2.6.3. Basophils and Mast Cells

Less than 1% of leukocytes are basophils, and they share morphological and functional similarities with mast cells. However, they are not redundant, as they present antigens (induction of TH2 cells) and release tryptase, histamine, chondroitin sulfate, leukotriene C4, leukotriene D4 and E4, and can induce anaphylaxis by IgG-mediated release of basophil-derived platelet-activating factor in mice [103]. In addition, they are a major source of IL-4 and IL-13, which can be induced in an IgE-dependent or independent manner [103]. Therefore, basophils may play a critical role in chronic inflammatory skin disease or allergic disorders, such as asthma and IgE-induced chronic allergic reactions [103]. Cyclic-AMP is one of the most effective inhibitors of basophil function and can therefore inhibit the stimulated release of histamine. Leukotriene and histamine release were attenuated by PDE4-I (rolipram, denbufylline, Ro 20-1724 and RP 73401), however, not by PDE1-I (8-methoxymethyl and IBMX), PDE3-I (siguazodan, SKF 94120 and SKF 95654) or PDE5-I (zaprinast) in human basophils [104]. Interestingly, human basophils express PDE4A and PDE4D, but little PDE4B or C mRNA or protein [105]. In contrast, human mast cells showed no effect of specific PDE4-I on the release on chemokines; accordingly, there is no evidence of any PDE4 in human mast cells [105].

#### 2.6.4. Macrophages

Macrophages play a key role in the innate immune system. Elevation of cAMP in macrophages can lead to suppression of receptor-mediated phagocytosis and reduction of inflammatory mediators via the PKA and Epac downstream pathways [106]. The PDE4-I Ro 20-1724 attenuated the release of TNF-α in murine macrophages after stimulation with the oxidant tert-butylhydroperoxide [107]. Human macrophages isolated from resected lungs showed a reduction of LPS-induced release of CCL2, 3 and 4 (C-C motif ligand), CXCL 10 (C-X-C motif ligand) and TNF-α when incubated with roflumilast or the active metabolite roflumilast N-oxide [108]. The inhibition of PDE4B resulted in downregulation of TNF-α and CCL3 and upregulation of the anti-inflammatory cytokine interleukin-1 receptor antagonist (IL-1Ra) in murine macrophages. However, the subtypes PDE4A and D were not involved in this response [109]. Interestingly CHF6001—an inhalable PDE4 I—reduced the number of macrophages in the sputum of COPD patients only at low doses, whereas neutrophil, eosinophil and lymphocyte count were not affected [110].

#### 2.6.5. Lymphocytes

In general, increased cAMP exerts inhibitory effects on lymphocytes, including cell cycle arrest and apoptosis. PDE3, PDE4 and PDE7 are the most abundant forms in these cells.

#### 2.6.6. B-Cells

Interestingly, high PDE4B expression is a marker for fatal outcome of diffuse large B-cell lymphoma (DLBCL) [111]. The increased levels of PDE4B in DLBCL may be responsible for the resistance to cAMP-induced apoptosis in these cells. The underlying mechanism was linked to the modulation of the B-cell receptor (BCR) and its downstream effectors, including phosphorylation of p85 and the activity of PI3K. This cAMP-specific inhibitory pathway was detected in mature B-cells as well as in DLBCL. Roflumilast (PDE4-I) increased survival and suppressed tumor burden in a murine B-cell lymphoma model [112]. Furthermore, PDE4-I restored the response to dexamethasone therapy in a glucocorticoid (GC)-resistant human B-cell lymphoma model [113]. Therefore, roflumilast was tested in a pilot phase Ib study in patients with relapsed/refractory B-cell lymphoma. Ten patients were included, and roflumilast could be administered safely and PI3K/AKT served as a marker. The authors postulated that roflumilast might have antitumor effects in human B-cell lymphoma. In acute lymphoblastic leukemia (ALL), pharmacologic inhibition of the PDE4 resulted in additional growth suppression [114,115]. Pentoxifylline (an unselective PDE-I) or placebo was tested in a phase 2 randomized study (NCT02451774, clinicaltrials.gov) with prednisone and chemotherapy in pediatric patients with acute lymphoblastic leukemia. It is postulated that PDE4B (overexpression) was responsible for ALL relapse because of inhibition of the glucocorticoid response, therefore causing the therapy to fail [115].

#### 2.6.7. T Cells

PDE also act as a critical regulator of T-cell function. PDE3, PDE4 and PDE7 are the predominant PDEs expressed in isolated CD4+ and CD8+ T lymphocytes [116]. PDE4B was the dominant PDE4 form in isolated activated murine CD4+ T lymphocytes, both in vivo and in vitro [116]. T-cell function was normal in PDE4A−/− and PDE4D−/− mice, whereas PDE4B−/− mice revealed a defect in T-cell proliferation [117]. PDE4 activity is less than 50% of overall PDE activity in T cells [118]. Therefore, the PDE4 independent regulation of cAMP levels in T cells may mean PDE4 inhibitors are not a top focus for clinical implications for T cells. However, INF-α reduces cAMP in T cells (CD4+CD25highFoxp+ regulatory T cells) by activation of PDE4 from the MER/ERK-mediated pathway. This leads to deactivation of the suppressive function of these human T cells [119]. Rolipram, however, weakly suppressed T-cell proliferation [118,120], and the release of IL-2, IL-5 and IFN-γ was reduced by piclamilast (RP73401/PDE4-I) in human CD4+ T cells.

#### 2.6.8. Natural Killer Cells

PDE4-I displayed a distinct inhibitory pattern to NK-cell responses [121]. Increased cAMP reduced the ability of natural killer (NK) cells to bind with target cells and decreased their cytotoxicity [111]. The PDE4-I rolipram, zardaverine and ibudilast showed inhibitory effects to exocytosis, TNF and IFN-γ production, and reduced anti-leukocyte functional antigen -1 production in NK cells [121]. To date, there are no data available about PDE4 subtype expression in these cells.

#### 2.6.9. Other Immune-Modulation Cell Types

Other cell types that are not primarily considered to be a part of the immune system are also involved in the immune response and cytokine release. These pathways can also be cAMP-dependent.

##### Fibroblasts

Rheumatoid arthritis (RA) is a chronic autoimmune and inflammatory disease. IL-18 plays a pivotal role in RA, because it induces INF-γ production and release. Furthermore, IL-18 triggers inflammatory responses by activating the NF-κB pathway. IL-18 overexpression is shown in RA, and can therefore induce proliferation of fibroblast-like synoviocytes (FS). Activated FS can release proinflammatory cytokines, chemokines and extracellular-matrix degradation enzymes. In vitro, roflumilast decreased IL-18 induced oxidative stress (ROS and MDA) in FS. Roflumilast reduced the secretion of MMP3, MMP13, CCL5, CXCL9 and CXCL10 in FS and had an inhibitory effect on NF-κB and AP-1 [122].

In dermal fibroblasts, PDE4 reduction of cAMP induced cell proliferation, extracellular matrix synthesis and downregulation of apoptosis. TGF-β1 is considered a key player for the profibrotic effects by small mothers against decapentaplegic (Smad) family members, Smad2 and 3. In human cultured skin, fibroblast apremilast inhibits profibrotic activity and blocks the TGF-β1-activated Smad and Erk 1/2 pathways in these cells [123].

In systemic sclerosis (SC), and particularly in patients with inflammation-driven fibrosis, PDE4-I could have disease-modifying antifibrotic effects. In a preclinical mice model of SC, PDE4-I (rolipram and apremilast) reduced and reversed chronic fibrosis by reducing inflammatory cytokines through reduction of the release of M2-macrophages [124].

##### Keratinocytes

PDE4 isoforms are expressed in keratinocytes. Proliferation and apoptosis can be induced in human keratinocytes by β-amyloid, which acts by the nerve growth factor (NGF) receptor CD271. PDE4-I (apremilast) reduced β-amyloid-induced cAMP degradation in these cells, and therefore reduced proliferation and apoptosis, and normalized IL-10 and decreased IL-1β and IL-8 levels. PDE4-I [70] also decreased additional TNF-α expression.

##### Epithelial Cells

Pulmonary epithelial cells secrete proinflammatory cyto- and chemokines. These include IL-8, TNF-α, GM-CSF and MCP-1. Thus, ensifentrine (PDE4 and 3 inhibitor) reduced their secretion in bronchial epithelial cells. This mechanism is exclusively driven by PDE4 [125]. In human alveolar epithelial cells (A549; stimulated with cigarette smoke and LPS), roflumilast and its active metabolite roflumilast N-oxide reduced IL-8 and MCP-1 release and the secretion of CXCL1 [126]. TNF-α-induced eotaxin protein expression—the strongest chemotactic agents for eosinophils—was reduced by coincubation with roflumilast in a human bronchial epithelial cell line (BEAS-2B) [127]. Cilominast caused a basal reduction of IL-6 and GMCSF in primary bronchial epithelial cells harvested from lung allograft recipients; interestingly, vascular endothelial growth factor (VEGF) was not affected [128].

### 2.7. Cancer

In view of the considerations outlined above, the potential role of PDE4-I in cancer progression and/or therapy must be addressed (see review [129]). Increased cAMP levels can induce apoptosis, arrest cell growth and reduce cell migration [130]. The underlying hypothesis is that PDE4 is upregulated in cancer cells, as seen in hematologic (T- and B-leukemic cells), lung, colon and hepatocellular cancer, glioblastoma, medulloblastoma, glioma and melanoma [129,130,131]. On the other hand, it has also been reported that PDE4 can be downregulated, e.g., in chronic lymphocytic leukemia and breast and prostate cancer [129]. Therefore, the use of PDE4-I in cancer therapy has to be discussed with reservations. There are two major concerns for the use of PDE4-I or even unselected PDE-I: First, there is a distinct pattern of regulation and localization of the different PDE4 subtypes in cancer cells. Regarding prostate cancer, PDE4A is downregulated, PDE4D increased overall, but PDE4D7 is upregulated when androgen-sensitive and downregulated if not [129]. The second concern for the use of PDE4-I for cancer therapy might be the immunosuppressive pattern of PDE4-I, which may worsen the prognosis. On the other hand, PDE4-I in cancer therapy may help to enhance radiation and/or chemotherapy effects [132] or can serve as a supportive therapy to refractory-chemotherapeutic treatment. However, to our knowledge, PDE4-I was only tested in a human trial for B-cell lymphoma therapy (see Section 2.6.6). In view of this, there is definitely a need for the development of highly specific PDE4-I before its use can be discussed in cancer therapy.

## 3. Outlook and Perspectives of Phosphodiesterase-4-Inhibition

In view of these observations, there is enormous therapeutic potential for phosphodiesterase-4 inhibition in various diseases, as shown in Table 1. However, since many tissues express phosphodiesterase-4, there is also space for significant side effects. Therefore, one problem that remains to be solved is the achievement of tissue- and cell-specificity for the desired mechanisms and therapeutic aims. This should be part of future pharmacological research. Nevertheless, the use of phosphodiesterase-4 inhibitors may give way to a breakthrough in the additive therapy for systemic inflammation in sepsis, since, at least in experimental models, there has been a significant effect to modulate aberrant immune responses, stabilize microvascular endothelial barrier function and to restore microcirculatory flow. All of these problems remain unresolved and may be overcome by the therapeutic use of phosphodiesterase-4 inhibitors. Although there are still many obstacles, the current data should strongly encourage researchers to pursue the therapeutic potential of phosphodiesterase-4 inhibition.

## Figures and Tables

**Table 1:** List of PDE-4-I named in the manuscript with human clinical trials numbers (data collected from www.clinicaltrial.gov, accessed on 30 December 2021).

Name	Disease	Clinical Trials	
Apremilast	Psoriasis, Psoriasis-arthritis, Discoid Lupus Erythematosus, Atopic Dermatitis, Lichen Planus of Vulva, Acne Conglobata	NCT00708916, NCT01393158, NCT03656666, NCT04161456, NCT01307423, NCT01212770, NCT01212757, NCT01172938	
CDP840		none	
CHF6001	COPD, Asthma	NCT02986321, NCT04756960, NCT04739774, NCT03004495, NCT03004417, NCT01689571, NCT04636801, NCT04636814	
Cilomilast	Pulmonary Disease, Chronic Obstructive	NCT00103922	
Crisaborole	Atopic Dermatitis; Eczema; Seborrheic Dermatitis; Alopecia Areata; Stasis Dermatitis; Psoriasis	NCT03233529, NCT04498403, NCT04040192, NCT03832010, NCT03539601, NCT03233529, NCT04214197, NCT04360187, NCT03567980, NCT03868098, NCT04299503, NCT04091087, NCT03760042, NCT03356977, NCT03260595, NCT04008784 NCT03954158, NCT01652885, NCT04800185, NCT01300052, NCT01602341, NCT02118792, NCT02118766, NCT01301508, NCT04194814, NCT03770858, NCT03645057, NCT00759161, NCT00755196, NCT01029405, NCT01258088, NCT00763204, NCT00762658	
Denbufylline		none	
DRM02	Rosacea, Atopic Dermatitis, Psoriasis	NCT01993446, NCT01993420, NCT01993433	
E6005/RVT-501		none	
Ibudilast	Alcohol Use Disorders, Methamphetamine-dependence, Medication Overuse Headache, Migraine Headache, Amyotrophic Lateral Sclerosis, Opioid Abuse, Glioblastoma, Amyotrophic Lateral Sclerosis, Pneumonia, Vira, Multiple Sclerosis, Myelopathy, Spinal Cord Diseases	NCT02025998, NCT03594435, NCT03341078, NCT01317992, NCT01389193, NCT01860807, NCT04057898, NCT01740414, NCT03782415, NCT02238626, NCT03489850, NCT01217970, NCT02714036, NCT04054206, NCT03533387, NCT04429555, NCT01982942, NCT04631471	
LEO 29102	Psoriasis Vulgaris, Atopic Dermatitis	NCT00891709, NCT01447758, NCT01005823, NCT00958516, NCT01037881, NCT00875277, NCT01466478, NCT01423656	
Nitraquazone		none	
OPA-15406/MM36.	Atopic Dermatitis	NCT02914548, NCT03908970, NCT03911401, NCT03961529, NCT03018691, NCT02334787, NCT02068352, NCT01702181	
Piclamilast		none	
Rolipram		NCT00369798,NCT01215552,NCT00250172, NCT01602900, NCT00011375	
Roflumilast	COPD, Blood sugar and Insulin, Major Depressive Disorder, Asthma, post Stroke, Obesity, Alzheimer’s Disease, Bronchiectasis, Non-cystic Fibrosis, Dermatitis, Diabetes Mellitus Type 2, Respiratory Distress Syndrome, Psoriasis, Obesity, Polycystic Ovary Syndrome, Dementia, Mild Cognitive Impairment, Sarcoidosis, Diabetic Nephropathies, Lymphoma (B-Cell), Schizophrenia, Atopic Dermatitis, Nonalcoholic Steatohepatitis, Major Depressive Disorder, Allergy, Seborrheic Dermatitis,	NCT04108377,NCT02363335, NCT04751071,NCT00246935,NCT00242294, NCT00242307, NCT00246922,NCT04854811, NCT01862029,NCT02835716, NCT04636814, NCT02386761, NCT01703052, NCT02119247, NCT01730404, NCT02986321, NCT02018432, NCT04322929, NCT01745848, NCT01354782, NCT00424268, NCT00430729, NCT01365533, NCT01329029, NCT00746382, NCT04069312, NCT04122547, NCT01630200, NCT01509677, NCT01973998, NCT00163475, NCT02068456, NCT01443845, NCT00076076, NCT00076089, NCT00313209, NCT01140542, NCT04744090, NCT03428334, NCT01473758, NCT04549870, NCT02037672, NCT00746434, NCT00297102, NCT00297115, NCT00242320, NCT01433666, NCT00062582, NCT00163527, NCT04658654, NCT01830959, NCT04090294, NCT03381573, NCT02451540, NCT02187250, NCT03988816, NCT01313494, NCT04755946, NCT02097992, NCT01849341, NCT04369547, NCT04800172, NCT03458546, NCT03073798, NCT01765192, NCT02079844, NCT01701934, NCT01856764, NCT00940329, NCT02015767, NCT01888952, NCT02165826, NCT01664624, NCT01595750, NCT02051335, NCT02187926, NCT00073177, NCT00108823, NCT01572948, NCT01480661, NCT02671942, NCT04636814, NCT01285180, NCT05028582, NCT01285167, NCT01580748, NCT04286607, NCT04773587, NCT04211363, NCT04211389, NCT04973228, NCT04773600, NCT04845620, NCT04804605	
Ro 20-1724		none	
RP 73401		none	
Xanthine nucleus		none	
Zardaverine		none	

## References

[1] Baillie G.S. (2009). Compartmentalized signalling: Spatial regulation of cAMP by the action of compartmentalized phosphodiesterases. FEBS J..

[2] Beavo J.A., Brunton L.L. (2002). Cyclic nucleotide research—Still expanding after half a century. Nat. Rev. Mol. Cell Biol..

[3] Tibbo A.J., Baillie G.S. (2020). Phosphodiesterase 4B: Master regulator of brain signaling. Cells.

[4] Sanders O., Rajagopal L. (2020). Phosphodiesterase inhibitors for Alzheimer’s disease: A systematic review of clinical trials and epidemiology with a mechanistic rationale. J. Alzheimers Dis. Rep..

[5] Norman T.R., Judd F.K., Burrows G.D. (1992). New pharmacological approaches to the management of depression: From theory to clinical practice. Aust. N. Z. J. Psychiatry.

[6] Torphy T.J., Livi G.P., Balcarek J.M., White J.R., Chilton F.H., Undem B.J. (1992). Therapeutic potential of isozyme-selective phosphodiesterase inhibitors in the treatment of asthma. Adv. Second Messenger Phosphoprot. Res..

[7] Kumar N., Goldminz A.M., Kim N., Gottlieb A.B. (2013). Phosphodiesterase 4-targeted treatments for autoimmune diseases. BMC Med..

[8] Wang H., Gaur U., Xiao J., Xu B., Xu J., Zheng W. (2018). Targeting phosphodiesterase 4 as a potential therapeutic strategy for enhancing neuroplasticity following ischemic stroke. Int. J. Biol. Sci..

[9] Schafer P. (2012). Apremilast mechanism of action and application to psoriasis and psoriatic arthritis. Biochem. Pharmacol..

[10] Oldenburger A., Maarsingh H., Schmidt M. (2012). Multiple facets of cAMP signalling and physiological impact: cAMP compartmentalization in the lung. Pharmaceuticals.

[11] Omori K., Kotera J. (2007). Overview of PDEs and their regulation. Circ. Res..

[12] Pfeifer A., Kilic A., Hoffmann L.S. (2013). Regulation of metabolism by cGMP. Pharmacol. Ther..

[13] Schindler R.F., Brand T. (2016). The Popeye domain containing protein family—A novel class of cAMP effectors with important functions in multiple tissues. Prog. Biophys. Mol. Biol..

[14] Zambon A.C., Zhang L., Minovitsky S., Kanter J.R., Prabhakar S., Salomonis N., Vranizan K., Dubchak I., Conklin B.R., Insel P.A. (2005). Gene expression patterns define key transcriptional events in cell-cycle regulation by cAMP and protein kinase A. Proc. Natl. Acad. Sci. USA.

[15] Lehrke M., Kahles F., Makowska A., Tilstam P.V., Diebold S., Marx J., Stohr R., Hess K., Endorf E.B., Bruemmer D. (2015). PDE4 inhibition reduces neointima formation and inhibits VCAM-1 expression and histone methylation in an Epac-dependent manner. J. Mol. Cell. Cardiol..

[16] Wang H., Xu J., Lazarovici P., Quirion R., Zheng W. (2018). cAMP response element-binding protein (CREB): A possible signaling molecule link in the pathophysiology of schizophrenia. Front. Mol. Neurosci..

[17] Meyers J.A., Su D.W., Lerner A. (2009). Chronic lymphocytic leukemia and B and T cells differ in their response to cyclic nucleotide phosphodiesterase inhibitors. J. Immunol..

[18] Hernandez-Florez D., Valor L. (2016). Selective phosphodiesterase inhibitors: A new therapeutic option in inflammation and autoimmunity. Reumatol. Clin..

[19] Houslay M.D., Baillie G.S., Maurice D.H. (2007). cAMP-Specific phosphodiesterase-4 enzymes in the cardiovascular system: A molecular toolbox for generating compartmentalized cAMP signaling. Circ. Res..

[20] Houslay M.D., Adams D.R. (2003). PDE4 cAMP phosphodiesterases: Modular enzymes that orchestrate signalling cross-talk, desensitization and compartmentalization. Biochem. J..

[21] Zhang H.T., Zhao Y., Huang Y., Deng C., Hopper A.T., de Vivo M., Rose G.M., O’Donnell J.M. (2006). Antidepressant-like effects of PDE4 inhibitors mediated by the high-affinity rolipram binding state (HARBS) of the phosphodiesterase-4 enzyme (PDE4) in rats. Psychopharmacology.

[22] Palacios J.M., Beleta J., Segarra V. (1995). Second messenger systems as targets for new therapeutic agents: Focus on selective phosphodiesterase inhibitors. Farmaco.

[23] Li H., Zuo J., Tang W. (2018). Phosphodiesterase-4 inhibitors for the treatment of inflammatory diseases. Front. Pharmacol..

[24] Zhang H.T. (2009). Cyclic AMP-specific phosphodiesterase-4 as a target for the development of antidepressant drugs. Curr. Pharm. Des..

[25] Gehlert D.R., Dawson T.M., Yamamura H.I., Wamsley J.K. (1985). Quantitative autoradiography of [3H] forskolin binding sites in the rat brain. Brain Res..

[26] Poat J.A., Cripps H.E., Iversen L.L. (1988). Differences between high-affinity forskolin binding sites in dopamine-rich and other regions of rat brain. Proc. Natl. Acad. Sci. USA.

[27] Delhaye S., Bardoni B. (2021). Role of phosphodiesterases in the pathophysiology of neurodevelopmental disorders. Mol. Psychiatry.

[28] Fujita M., Richards E.M., Niciu M.J., Ionescu D.F., Zoghbi S.S., Hong J., Telu S., Hines C.S., Pike V.W., Zarate C.A. (2017). cAMP signaling in brain is decreased in unmedicated depressed patients and increased by treatment with a selective serotonin reuptake inhibitor. Mol. Psychiatry.

[29] Cherry J.A., Davis R.L. (1999). Cyclic AMP phosphodiesterases are localized in regions of the mouse brain associated with reinforcement, movement, and affect. J. Comp. Neurol..

[30] Takahashi M., Terwilliger R., Lane C., Mezes P.S., Conti M., Duman R.S. (1999). Chronic antidepressant administration increases the expression of cAMP-specific phosphodiesterase 4A and 4B isoforms. J. Neurosci..

[31] Zhu J., Mix E., Winblad B. (2001). The antidepressant and antiinflammatory effects of rolipram in the central nervous system. CNS Drug Rev..

[32] Zhao Y., Zhang H.T., O’Donnell J.M. (2003). Antidepressant-induced increase in high-affinity rolipram binding sites in rat brain: Dependence on noradrenergic and serotonergic function. J. Pharmacol. Exp. Ther..

[33] Richter W., Menniti F.S., Zhang H.T., Conti M. (2013). PDE4 as a target for cognition enhancement. Expert Opin. Ther. Targets.

[34] McGirr A., Lipina T.V., Mun H.S., Georgiou J., Al-Amri A.H., Ng E., Zhai D., Elliott C., Cameron R.T., Mullins J.G. (2016). Specific inhibition of phosphodiesterase-4B results in anxiolysis and facilitates memory acquisition. Neuropsychopharmacology.

[35] Wood M.A., Kaplan M.P., Park A., Blanchard E.J., Oliveira A.M., Lombardi T.L., Abel T. (2005). Transgenic mice expressing a truncated form of CREB-binding protein (CBP) exhibit deficits in hippocampal synaptic plasticity and memory storage. Learn Mem..

[36] Heckman P.R.A., Van Duinen M.A., Blokland A., Uz T., Prickaerts J., Sambeth A. (2018). Acute administration of roflumilast enhances sensory gating in healthy young humans in a randomized trial. Psychopharmacology.

[37] Blokland A., Van Duinen M.A., Sambeth A., Heckman P.R.A., Tsai M., Lahu G., Uz T., Prickaerts J. (2019). Acute treatment with the PDE4 inhibitor roflumilast improves verbal word memory in healthy old individuals: A double-blind placebo-controlled study. Neurobiol. Aging.

[38] Kroegel C., Foerster M. (2007). Phosphodiesterase-4 inhibitors as a novel approach for the treatment of respiratory disease: Cilomilast. Expert Opin. Investig. Drugs.

[39] Konrad F.M., Bury A., Schick M.A., Ngamsri K.C., Reutershan J. (2015). The unrecognized effects of phosphodiesterase 4 on epithelial cells in pulmonary inflammation. PLoS ONE.

[40] Billington C.K., Ojo O.O., Penn R.B., Ito S. (2013). cAMP regulation of airway smooth muscle function. Pulm. Pharmacol. Ther..

[41] Sayner S.L. (2011). Emerging themes of cAMP regulation of the pulmonary endothelial barrier. Am. J. Physiol. Lung Cell. Mol. Physiol..

[42] Barber R., Baillie G.S., Bergmann R., Shepherd M.C., Sepper R., Houslay M.D., Heeke G.V. (2004). Differential expression of PDE4 cAMP phosphodiesterase isoforms in inflammatory cells of smokers with COPD, smokers without COPD, and nonsmokers. Am. J. Physiol. Lung Cell. Mol. Physiol..

[43] Mackenzie K.F., Topping E.C., Bugaj-Gaweda B., Deng C., Cheung Y.F., Olsen A.E., Stockard C.R., High Mitchell L., Baillie G.S., Grizzle W.E. (2008). Human PDE4A8, a novel brain-expressed PDE4 cAMP-specific phosphodiesterase that has undergone rapid evolutionary change. Biochem. J..

[44] Millen J., MacLean M.R., Houslay M.D. (2006). Hypoxia-induced remodelling of PDE4 isoform expression and cAMP handling in human pulmonary artery smooth muscle cells. Eur. J. Cell Biol..

[45] Sachs B.D., Baillie G.S., McCall J.R., Passino M.A., Schachtrup C., Wallace D.A., Dunlop A.J., MacKenzie K.F., Klussmann E., Lynch M.J. (2007). p75 neurotrophin receptor regulates tissue fibrosis through inhibition of plasminogen activation via a PDE4/cAMP/PKA pathway. J. Cell Biol..

[46] Cheung Y.F., Kan Z., Garrett-Engele P., Gall I., Murdoch H., Baillie G.S., Camargo L.M., Johnson J.M., Houslay M.D., Castle J.C. (2007). PDE4B5, a novel, super-short, brain-specific cAMP phosphodiesterase-4 variant whose isoform-specifying N-terminal region is identical to that of cAMP phosphodiesterase-4D6 (PDE4D6). J. Pharmacol. Exp. Ther..

[47] Shepherd M., McSorley T., Olsen A.E., Johnston L.A., Thomson N.C., Baillie G.S., Houslay M.D., Bolger G.B. (2003). Molecular cloning and subcellular distribution of the novel PDE4B4 cAMP-specific phosphodiesterase isoform. Biochem. J..

[48] Engels P., Fichtel K., Lubbert H. (1994). Expression and regulation of human and rat phosphodiesterase type IV isogenes. FEBS Lett..

[49] Engels P., Sullivan M., Muller T., Lubbert H. (1995). Molecular cloning and functional expression in yeast of a human cAMP-specific phosphodiesterase subtype (PDE IV-C). FEBS Lett..

[50] Zuo H., Cattani-Cavalieri I., Musheshe N., Nikolaev V.O., Schmidt M. (2019). Phosphodiesterases as therapeutic targets for respiratory diseases. Pharmacol. Ther..

[51] Richter W., Jin S.L., Conti M. (2005). Splice variants of the cyclic nucleotide phosphodiesterase PDE4D are differentially expressed and regulated in rat tissue. Biochem. J..

[52] Luo J., Yang L., Yang J., Yang D., Liu B.C., Liu D., Liang B.M., Liu C.T. (2018). Efficacy and safety of phosphodiesterase 4 inhibitors in patients with asthma: A systematic review and meta-analysis. Respirology.

[53] Vogelmeier C.F., Criner G.J., Martinez F.J., Anzueto A., Barnes P.J., Bourbeau J., Celli B.R., Chen R., Decramer M., Fabbri L.M. (2017). Global strategy for the diagnosis, management, and prevention of chronic obstructive lung disease 2017 report. GOLD executive summary. Am. J. Respir. Crit. Care Med..

[54] Hansen G., Jin S., Umetsu D.T., Conti M. (2000). Absence of muscarinic cholinergic airway responses in mice deficient in the cyclic nucleotide phosphodiesterase PDE4D. Proc. Natl. Acad. Sci. USA.

[55] Jacob C., Leport M., Szilagyi C., Allen J.M., Bertrand C., Lagente V. (2002). DMSO-treated HL60 cells: A model of neutrophil-like cells mainly expressing PDE4B subtype. Int. Immunopharmacol..

[56] Manning C.D., Burman M., Christensen S.B., Cieslinski L.B., Essayan D.M., Grous M., Torphy T.J., Barnette M.S. (1999). Suppression of human inflammatory cell function by subtype-selective PDE4 inhibitors correlates with inhibition of PDE4A and PDE4B. Br. J. Pharmacol..

[57] Mauad T., Dolhnikoff M. (2008). Pathologic similarities and differences between asthma and chronic obstructive pulmonary disease. Curr. Opin. Pulm. Med..

[58] Welte T., Groneberg D.A. (2006). Asthma and COPD. Exp. Toxicol. Pathol..

[59] Guerra S. (2009). Asthma and chronic obstructive pulmonary disease. Curr. Opin. Allergy Clin. Immunol..

[60] Hogg J.C., Timens W. (2009). The pathology of chronic obstructive pulmonary disease. Annu. Rev. Pathol..

[61] Meurs H., Gosens R., Zaagsma J. (2008). Airway hyperresponsiveness in asthma: Lessons from in vitro model systems and animal models. Eur. Respir. J..

[62] Giembycz M.A., Maurice D.H. (2014). Cyclic nucleotide-based therapeutics for chronic obstructive pulmonary disease. Curr. Opin. Pharmacol..

[63] Dekkers B.G., Racke K., Schmidt M. (2013). Distinct PKA and Epac compartmentalization in airway function and plasticity. Pharmacol. Ther..

[64] Lugnier C., Al-Kuraishy H.M., Rousseau E. (2021). PDE4 inhibition as a therapeutic strategy for improvement of pulmonary dysfunctions in Covid-19 and cigarette smoking. Biochem. Pharmacol..

[65] El Tabaa M.M., El Tabaa M.M. (2020). New putative insights into neprilysin (NEP)-dependent pharmacotherapeutic role of roflumilast in treating COVID-19. Eur. J. Pharmacol..

[66] Giorgi M., Cardarelli S., Ragusa F., Saliola M., Biagioni S., Poiana G., Naro F., Massimi M. (2020). Phosphodiesterase Inhibitors: Could They Be Beneficial for the Treatment of COVID-19?. Int. J. Mol. Sci..

[67] Gottlieb A.B., Dann F. (2009). Comorbidities in patients with psoriasis. Am. J. Med..

[68] Gottlieb A.B., Matheson R.T., Menter A., Leonardi C.L., Day R.M., Hu C., Schafer P.H., Krueger J.G. (2013). Efficacy, tolerability, and pharmacodynamics of apremilast in recalcitrant plaque psoriasis: A phase II open-label study. J. Drugs Dermatol..

[69] Gottlieb A.B., Strober B., Krueger J.G., Rohane P., Zeldis J.B., Hu C.C., Kipnis C. (2008). An open-label, single-arm pilot study in patients with severe plaque-type psoriasis treated with an oral anti-inflammatory agent, apremilast. Curr. Med. Res. Opin..

[70] Pincelli C., Schafer P.H., French L.E., Augustin M., Krueger J.G. (2018). Mechanisms underlying the clinical effects of apremilast for psoriasis. J. Drugs Dermatol..

[71] Schafer P.H., Truzzi F., Parton A., Wu L., Kosek J., Zhang L.H., Horan G., Saltari A., Quadri M., Lotti R. (2016). Phosphodiesterase 4 in inflammatory diseases: Effects of apremilast in psoriatic blood and in dermal myofibroblasts through the PDE4/CD271 complex. Cell. Signal..

[72] Gooderham M., Papp K. (2015). Selective phosphodiesterase inhibitors for psoriasis: Focus on apremilast. BioDrugs.

[73] Lebwohl M.G., Papp K.A., Stein Gold L., Gooderham M.J., Kircik L.H., Draelos Z.D., Kempers S.E., Zirwas M., Smith K., Osborne D.W. (2020). Trial of roflumilast cream for chronic plaque psoriasis. N. Engl. J. Med..

[74] Dong C., Virtucio C., Zemska O., Baltazar G., Zhou Y., Baia D., Jones-Iatauro S., Sexton H., Martin S., Dee J. (2016). Treatment of skin inflammation with benzoxaborole phosphodiesterase inhibitors: Selectivity, cellular activity, and effect on cytokines associated with skin inflammation and skin architecture changes. J. Pharmacol. Exp. Ther..

[75] Guttman-Yassky E., Hanifin J.M., Boguniewicz M., Wollenberg A., Bissonnette R., Purohit V., Kilty I., Tallman A.M., Zielinski M.A. (2019). The role of phosphodiesterase 4 in the pathophysiology of atopic dermatitis and the perspective for its inhibition. Exp. Dermatol..

[76] Grewe S.R., Chan S.C., Hanifin J.M. (1982). Elevated leukocyte cyclic AMP-phosphodiesterase in atopic disease: A possible mechanism for cyclic AMP-agonist hyporesponsiveness. J. Allergy Clin. Immunol..

[77] Samrao A., Berry T.M., Goreshi R., Simpson E.L. (2012). A pilot study of an oral phosphodiesterase inhibitor (apremilast) for atopic dermatitis in adults. Arch. Dermatol..

[78] Paller A.S., Tom W.L., Lebwohl M.G., Blumenthal R.L., Boguniewicz M., Call R.S., Eichenfield L.F., Forsha D.W., Rees W.C., Simpson E.L. (2016). Efficacy and safety of crisaborole ointment, a novel, nonsteroidal phosphodiesterase 4 (PDE4) inhibitor for the topical treatment of atopic dermatitis (AD) in children and adults. J. Am. Acad. Dermatol..

[79] Bissonnette R., Pavel A.B., Diaz A., Werth J.L., Zang C., Vranic I., Purohit V.S., Zielinski M.A., Vlahos B., Estrada Y.D. (2019). Crisaborole and atopic dermatitis skin biomarkers: An intrapatient randomized trial. J. Allergy Clin. Immunol..

[80] Gao T., Ji Y., Wang Y. (2021). The effects of dialysis modality choice on cognitive functions in patients with end-stage renal failure: A protocol for systematic review and meta-analysis. Medicine.

[81] Yan H., Xu J., Xu Z., Yang B., Luo P., He Q. (2021). Defining therapeutic targets for renal fibrosis: Exploiting the biology of pathogenesis. Biomed. Pharmacother..

[82] Nio Y., Ookawara M., Yamasaki M., Hanauer G., Tohyama K., Shibata S., Sano T., Shimizu F., Anayama H., Hazama M. (2020). Ameliorative effect of phosphodiesterase 4 and 5 inhibitors in deoxycorticosterone acetate-salt hypertensive uni-nephrectomized KKA(y) mice. FASEB J..

[83] Xu M., Yu X., Meng X., Huang S., Zhang Y., Zhang A., Jia Z. (2020). Inhibition of PDE4/PDE4B improves renal function and ameliorates inflammation in cisplatin-induced acute kidney injury. J. Physiol. -Ren. Physiol..

[84] Mammadov E., Aridogan I.A., Izol V., Acikalin A., Abat D., Tuli A., Bayazit Y. (2012). Protective effects of phosphodiesterase-4-specific inhibitor rolipram on acute ischemia-reperfusion injury in rat kidney. Urology.

[85] Wollborn J., Siemering S., Steiger C., Buerkle H., Goebel U., Schick M.A. (2019). Phosphodiesterase-4 inhibition reduces ECLS-induced vascular permeability and improves microcirculation in a rodent model of extracorporeal resuscitation. Am. J. Physiol. Heart Circ. Physiol..

[86] Xu X., Liao L., Hu B., Jiang H., Tan M. (2020). Roflumilast, a phosphodiesterases-4 (PDE4) inhibitor, alleviates sepsisinduced acute kidney injury. Med. Sci. Monit..

[87] Gorur S., Celik S., Hakverdi S., Aslantas O., Erdogan S., Aydin M., Ocak S., Namik Kiper A. (2008). Preventive effect of rolipram, a phosphodiesterase 4 enzyme inhibitor, on oxidative renal injury in acute ascending pyelonephritis model in rats. Urology.

[88] Holthoff J.H., Wang Z., Patil N.K., Gokden N., Mayeux P.R. (2013). Rolipram improves renal perfusion and function during sepsis in the mouse. J. Pharmacol. Exp. Ther..

[89] Fischmeister R. (2006). Is cAMP good or bad? Depends on where it is made. Circ. Res..

[90] Schlegel N., Waschke J. (2014). cAMP with other signaling cues converges on Rac1 to stabilize the endothelial barrier—A signaling pathway compromised in inflammation. Cell Tissue Res..

[91] Radeva M.Y., Kugelmann D., Spindler V., Waschke J. (2014). PKA compartmentalization via AKAP220 and AKAP12 contributes to endothelial barrier regulation. PLoS ONE.

[92] Schick M.A., Wunder C., Wollborn J., Roewer N., Waschke J., Germer C.T., Schlegel N. (2012). Phosphodiesterase-4 inhibition as a therapeutic approach to treat capillary leakage in systemic inflammation. J. Physiol..

[93] Schlegel N., Baumer Y., Drenckhahn D., Waschke J. (2009). Lipopolysaccharide-induced endothelial barrier breakdown is cyclic adenosine monophosphate dependent in vivo and in vitro. Crit. Care Med..

[94] Flemming S., Schlegel N., Wunder C., Meir M., Baar W., Wollborn J., Roewer N., Germer C.T., Schick M.A. (2014). Phosphodiesterase 4 inhibition dose dependently stabilizes microvascular barrier functions and microcirculation in a rodent model of polymicrobial sepsis. Shock.

[95] Sanz M.J., Cortijo J., Taha M.A., Cerda-Nicolas M., Schatton E., Burgbacher B., Klar J., Tenor H., Schudt C., Issekutz A.C. (2007). Roflumilast inhibits leukocyte-endothelial cell interactions, expression of adhesion molecules and microvascular permeability. Br. J. Pharmacol..

[96] Wang Y.J., Jiang Y.L., Tang H.F., Zhao C.Z., Chen J.Q. (2010). Zl-n-91, a selective phosphodiesterase 4 inhibitor, suppresses inflammatory response in a COPD-like rat model. Int. Immunopharmacol..

[97] Kubo S., Kobayashi M., Iwata M., Takahashi K., Miyata K., Shimizu Y. (2011). Disease-modifying effect of ASP3258, a novel phosphodiesterase type 4 inhibitor, on subchronic cigarette smoke exposure-induced lung injury in guinea pigs. Eur. J. Pharmacol..

[98] Grootendorst D.C., Gauw S.A., Verhoosel R.M., Sterk P.J., Hospers J.J., Bredenbroker D., Bethke T.D., Hiemstra P.S., Rabe K.F. (2007). Reduction in sputum neutrophil and eosinophil numbers by the PDE4 inhibitor roflumilast in patients with COPD. Thorax.

[99] Hatzelmann A., Schudt C. (2001). Anti-inflammatory and immunomodulatory potential of the novel PDE4 inhibitor roflumilast in vitro. J. Pharmacol. Exp. Ther..

[100] Martorana P.A., Lunghi B., Lucattelli M., De Cunto G., Beume R., Lungarella G. (2008). Effect of roflumilast on inflammatory cells in the lungs of cigarette smoke-exposed mice. BMC Pulm. Med..

[101] Schmidt M., Dekker F.J., Maarsingh H. (2013). Exchange protein directly activated by cAMP (epac): A multidomain cAMP mediator in the regulation of diverse biological functions. Pharmacol. Rev..

[102] Jin S.L., Goya S., Nakae S., Wang D., Bruss M., Hou C., Umetsu D., Conti M. (2010). Phosphodiesterase 4B is essential for T(H)2-cell function and development of airway hyperresponsiveness in allergic asthma. J. Allergy Clin. Immunol..

[103] Ito Y., Satoh T., Takayama K., Miyagishi C., Walls A.F., Yokozeki H. (2011). Basophil recruitment and activation in inflammatory skin diseases. Allergy.

[104] Weston M.C., Anderson N., Peachell P.T. (1997). Effects of phosphodiesterase inhibitors on human lung mast cell and basophil function. Br. J. Pharmacol..

[105] Eskandari N., Tashrifi F., Bastan R., Andalib A., Yousefi Z., Peachell P.T. (2016). Cyclic nucleotide phosphodiesterase isoforms in human basophils and mast cells. Int. J. Immunopathol. Pharmacol..

[106] Serezani C.H., Ballinger M.N., Aronoff D.M., Peters-Golden M. (2008). Cyclic AMP: Master regulator of innate immune cell function. Am. J. Respir. Cell Mol. Biol..

[107] Brown D.M., Hutchison L., Donaldson K., MacKenzie S.J., Dick C.A., Stone V. (2007). The effect of oxidative stress on macrophages and lung epithelial cells: The role of phosphodiesterases 1 and 4. Toxicol. Lett..

[108] Buenestado A., Grassin-Delyle S., Guitard F., Naline E., Faisy C., Israel-Biet D., Sage E., Bellamy J.F., Tenor H., Devillier P. (2012). Roflumilast inhibits the release of chemokines and TNF-alpha from human lung macrophages stimulated with lipopolysaccharide. Br. J. Pharmacol..

[109] Lai C.-R., Lo H.-C., Chen Y.-L., Yang J.-X., Ding S.-L., Hsu H.-H., Conti M., Wu C.-P., Catherine Jin S. (2015). Phosphodiesterase 4B is essential for lipopolysaccharide-induced CC chemokine ligand 3 production in mouse macrophages. J. Med. Sci..

[110] Singh D., Beeh K.M., Colgan B., Kornmann O., Leaker B., Watz H., Lucci G., Geraci S., Emirova A., Govoni M. (2019). Effect of the inhaled PDE4 inhibitor CHF6001 on biomarkers of inflammation in COPD. Respir. Res..

[111] Shipp M.A., Ross K.N., Tamayo P., Weng A.P., Kutok J.L., Aguiar R.C., Gaasenbeek M., Angelo M., Reich M., Pinkus G.S. (2002). Diffuse large B-cell lymphoma outcome prediction by gene-expression profiling and supervised machine learning. Nat. Med..

[112] Suhasini A.N., Wang L., Holder K.N., Lin A.P., Bhatnagar H., Kim S.W., Moritz A.W., Aguiar R.C.T. (2016). A phosphodiesterase 4B-dependent interplay between tumor cells and the microenvironment regulates angiogenesis in B-cell lymphoma. Leukemia.

[113] Kim S.W., Rai D., Aguiar R.C. (2011). Gene set enrichment analysis unveils the mechanism for the phosphodiesterase 4B control of glucocorticoid response in B-cell lymphoma. Clin. Cancer Res..

[114] Ogawa R., Streiff M.B., Bugayenko A., Kato G.J. (2002). Inhibition of PDE4 phosphodiesterase activity induces growth suppression, apoptosis, glucocorticoid sensitivity, p53, and p21(WAF1/CIP1) proteins in human acute lymphoblastic leukemia cells. Blood.

[115] Yang J.J., Cheng C., Devidas M., Cao X., Campana D., Yang W., Fan Y., Neale G., Cox N., Scheet P. (2012). Genome-wide association study identifies germline polymorphisms associated with relapse of childhood acute lymphoblastic leukemia. Blood.

[116] Dong H., Zitt C., Auriga C., Hatzelmann A., Epstein P.M. (2010). Inhibition of PDE3, PDE4 and PDE7 potentiates glucocorticoid-induced apoptosis and overcomes glucocorticoid resistance in CEM T leukemic cells. Biochem. Pharmacol..

[117] Vang A.G., Ben-Sasson S.Z., Dong H., Kream B., DeNinno M.P., Claffey M.M., Housley W., Clark R.B., Epstein P.M., Brocke S. (2010). PDE8 regulates rapid Teff cell adhesion and proliferation independent of ICER. PLoS ONE.

[118] Peter D., Jin S.L., Conti M., Hatzelmann A., Zitt C. (2007). Differential expression and function of phosphodiesterase 4 (PDE4) subtypes in human primary CD4+ T cells: Predominant role of PDE4D. J. Immunol..

[119] Bacher N., Raker V., Hofmann C., Graulich E., Schwenk M., Baumgrass R., Bopp T., Zechner U., Merten L., Becker C. (2013). Interferon-alpha suppresses cAMP to disarm human regulatory T cells. Cancer Res..

[120] Jung S., Zielasek J., Kollner G., Donhauser T., Toyka K., Hartung H.P. (1996). Preventive but not therapeutic application of Rolipram ameliorates experimental autoimmune encephalomyelitis in Lewis rats. J. Neuroimmunol..

[121] Theorell J., Gustavsson A.L., Tesi B., Sigmundsson K., Ljunggren H.G., Lundback T., Bryceson Y.T. (2014). Immunomodulatory activity of commonly used drugs on Fc-receptor-mediated human natural killer cell activation. Cancer Immunol. Immunother..

[122] Zhong B., Guo S., Yang Z., Han L., Du J., Chen J., Dun X., Wang G. (2021). Roflumilast reduced the IL-18-induced inflammatory response in fibroblast-like synoviocytes (FLS). ACS Omega.

[123] Cutolo M., Soldano S., Montagna P., Martinelli G., Tardito S., Corallo C., Giordano N., Tavilla P., Cozzani E., Parodi A. (2020). Apremilast interferes with the TGFbeta1-induced transition of human skin fibroblasts into profibrotic myofibroblasts: In vitro study. Rheumatology.

[124] Maier C., Ramming A., Bergmann C., Weinkam R., Kittan N., Schett G., Distler J.H.W., Beyer C. (2017). Inhibition of phosphodiesterase 4 (PDE4) reduces dermal fibrosis by interfering with the release of interleukin-6 from M2 macrophages. Ann. Rheum. Dis..

[125] Turner M.J., Dauletbaev N., Lands L.C., Hanrahan J.W. (2020). The Phosphodiesterase inhibitor ensifentrine reduces production of proinflammatory mediators in well differentiated bronchial epithelial cells by inhibiting PDE4. J. Pharmacol. Exp. Ther..

[126] Victoni T., Gleonnec F., Lanzetti M., Tenor H., Valenca S., Porto L.C., Lagente V., Boichot E. (2014). Roflumilast N-oxide prevents cytokine secretion induced by cigarette smoke combined with LPS through JAK/STAT and ERK1/2 inhibition in airway epithelial cells. PLoS ONE.

[127] Paplinska M., Chazan R., Grubek-Jaworska H. (2011). Effect of phoshpodiesterase 4 (PDE4) inhibibtors on eotaxin expression in humen bronchial epithelial cells. J. Physiol. Pharmacol..

[128] Murphy D.M., Ward C., Forrest I.A., Pritchard G., Jones D., Stovold R., Fisher A.J., Cawston T.E., Lordan J.L., Corris P.A. (2006). The phosphodiesterase type IV inhibitor cilomilast decreases pro-inflammatory cytokine production from primary bronchial epithelial cells in lung transplantation patients. J. Heart Lung Transplant..

[129] Hsien Lai S., Zervoudakis G., Chou J., Gurney M.E., Quesnelle K.M. (2020). PDE4 subtypes in cancer. Oncogene.

[130] Massimi M., Ragusa F., Cardarelli S., Giorgi M. (2019). Targeting Cyclic AMP Signalling in Hepatocellular Carcinoma. Cells.

[131] Marko D., Pahlke G., Merz K.H., Eisenbrand G. (2000). Cyclic 3′,5′-nucleotide phosphodiesterases: Potential targets for anticancer therapy. Chem. Res. Toxicol..

[132] Goldhoff P., Warrington N.M., Limbrick D.D., Hope A., Woerner B.M., Jackson E., Perry A., Piwnica-Worms D., Rubin J.B. (2008). Targeted inhibition of cyclic AMP phosphodiesterase-4 promotes brain tumor regression. Clin. Cancer Res..

